# The Development of Binocular Suppression in Infants

**DOI:** 10.3389/fpsyg.2020.558871

**Published:** 2020-10-23

**Authors:** Jiale Yang, So Kanazawa, Masami K. Yamaguchi

**Affiliations:** ^1^Research and Development Initiative, Chuo University, Tokyo, Japan; ^2^Department of Psychology, Japan Women’s University, Kanagawa, Japan; ^3^Department of Psychology, Chuo University, Tokyo, Japan

**Keywords:** binocular suppression, infant, preferential looking paradigm, visual development, continuous flash suppression

## Abstract

Little is known about the time of development of binocular suppression. In the present study, we evaluated the emergence of binocular suppression in infants by using continuous flash suppression (CFS, Tsuchiya and Koch, 2005). In our experiment, one eye of infants was presented with a static face image at one side of the screen, while another eye was presented with dynamic Mondrian patterns in full screen. Adult observers confirmed that the static face image was consciously repressed by the changing Mondrian patterns. If binocular suppression was functional, the infants would not perceive the face and thus would not show any preference in the experiment. However, if binocular suppression in the infants was not yet acquired, they would perceive the face and the Mondrian patterns at the same time and would thus show preference for the side where the face was presented. The results showed that infants aged 2–3 months, but not those aged 4–5 months, detected the position of the face. Furthermore, this detection was not due to weak contrast sensitivity to the dynamic Mondrian mask. These results indicated that the immature binocular visual system may perceive different images from different eyes simultaneously and that infants may lose this ability after establishing binocular suppression at 4–5 months of age.

## Introduction

Several studies on binocular vision in infants have found that most infants have an average stereopsis onset between the ages of 2 and 4 months (Fox et al., 1980; Held et al., 1980; Petrig et al., 1981). Developmental stereopsis has been investigated by measuring the emergence of the sensitivity to binocular disparity (Held et al., 1980; Petrig et al., 1981; Skarf et al., 1993; Birch and Petrig, 1996; Kavšek, 2013a). In these studies, two paradigms have been applied, namely, the measurement of visual evoked potentials (VEPs) and the measurement of looking time to the stimuli containing or without horizontal disparity information. These studies suggested that sensitivity to horizontal disparity emerges after 3 months of age.

Binocular rivalry, another perceptual phenomenon of binocular vision, has been investigated by testing whether the infant can discriminate between fusible and rivalrous stimuli (Birch et al., 1985; Shimojo et al., 1986; Gwiazda et al., 1989; Thorn et al., 1994; Brown and Miracle, 2003; Kavšek, 2013b). In this method, two stimuli were presented dichoptically to infants. One stimulus consisted of an interocularly identical pattern (fusible stimulus) and the other of an interocularly different pattern (rivalrous stimulus). The forced-choice preferential looking (FPL) method (e.g., Teller, 1979) was used to examine whether the infants showed a preference for certain stimuli, which is regarded as discrimination between the fusible and rivalrous stimuli. These studies reveal that the infants looked longer at the fusional stimuli than the rivalrous ones after an average age of 2 months, suggesting that binocular rivalry emerges at a similar period as stereopsis.

These previous studies using preferential looking methods to test the discrimination between fusible and rivalrous patterns depend on whether infants have a spontaneous preference for the fusible pattern or rivalry pattern. If infants show a preference for certain stimuli, this indicates that the infants can detect fusible stimuli from rivalrous stimuli. However, a null result in the younger group cannot be interpreted as that these infants do not perceive the binocular rivalry, because it is possible that these infants would show no spontaneous preference for the fusible pattern or rivalrous pattern. Therefore, the emergence of preference shown by previous studies could not be considered as the developmental onset of the binocular rivalry.

In the present study, we evaluated the development of binocular rivalry in infants by using continuous flash suppression (CFS, Tsuchiya and Koch, 2005), which does not depend on the spontaneous preference for either the fusible or the rivalrous pattern. Hence, it can avoid a null result as commonly observed in prior studies, which is hard to interpret with respect to whether infants do show binocular rivalry or not. In the CFS procedure, a target stimulus is continuously presented to one eye, while continuous flashing of random Mondrian images is presented to the other eye. This CFS prevents participants from seeing the target image. Different from classical binocular rivalry, the target can be completely suppressed for over 1 min by using the CFS paradigm (Tsuchiya and Koch, 2005). Thanks to this long suppression time, CFS allows researchers to manipulate conscious perception. Therefore, CFS is used as a powerful tool in the aspect of consciousness studies (for a review, see Axelrod et al., 2015). For instance, a recent adult study showed that images of dominant and untrustworthy faces, compared to neutral faces, took a longer time to emerge to awareness, suggesting that information about personality characteristics can be processed outside of awareness (Stewart et al., 2012). If we could demonstrate that CFS can be utilized in infants successfully, this would not only give us an opportunity to investigate binocular suppression in infants but also indicate that CFS can be used in consciousness studies in infants.

**FIGURE 1:**
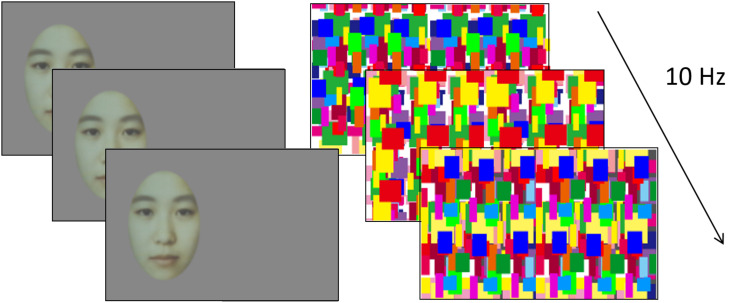
Example of the experiment stimulus in the experiment 1. In each trial, dynamic Mondrian masks were presented to one eye while a face stimulus was presented to the other eye. The position of the face stimulus was either left or right. The face stimulus was generated by averaging different 20 women’s faces.

In the present study, one eye of each infant was presented with a static face image at one side of the screen, while the other eye was presented with dynamic Mondrian patterns over the entire screen (Figure 1). If binocular suppression has developed, the infants would not perceive the face similarly as adults and thus would show no face preference in the experiment. If the infants had not acquired binocular suppression yet, they would perceive the face and the Mondrian patterns simultaneously and would thus show preference for the face. We used the face as a target because a visible face elicits reliable attentional biases toward it even in newborns (Johnson et al., 1991; Mondloch et al., 1999; Cassia et al., 2004; Di Giorgio et al., 2012). If the face stimulus was visible for infants in present study, the infants would perceive a face mixed with dynamic Mondrian patterns. We hypothesized that this “mixed face” would attract the infants’ attention and give rise to preferential looking toward the side where the “mixed face” was located.

## Experiment 1

In the first experiment, we investigated the development of binocular suppression in infants by using CFS.

## Materials and Methods

### Participants

Fifteen infants aged 2–3 months (7 male, 8 female, mean age = 73.6 days, and age range 51–98 days) and 15 infants aged 4–5 months (6 male, 9 female, mean age = 137.8 days, and age range 105–161 days) were included in the study. Although 25 other infants were tested in Experiment 1, they were excluded from the analysis because of fussiness (*n* = 7), side bias of more than 90% (*n* = 16), or technical problems (*n* = 2). All infants were recruited through advertisements in the newspaper and were full-term at birth and healthy at the time of the experiment. Ethical approval for this study was obtained from the ethical committee at Chuo University. Moreover, the experiments were conducted according to the principles of the Helsinki declaration. Written informed consent was obtained from the parents of the infants prior to the start of the experiment.

### Apparatus

During the experiment, each infant sat on his or her parent’s lap in the experimental booth. A 22-inch three-dimensional liquid-crystal display (3D-LCD) monitor (ZM-M220W; Zalman Tech Co Ltd.) that displayed all the stimuli was placed in front of the infant, at a distance of about 40 cm. Infants wore circular 3D glasses to watch the stimuli during the experiment. The center of the monitor was at the infant’s eye level, and its resolution was set at 1,680 × 1,050 pixels. The infant’s looking behavior was recorded through a video camera set under the monitor. Behind the experimental booth, the infant’s behavior was also observed via a TV monitor.

### Stimuli

Two different images were dichoptically presented to both eyes of the infants (Figure 1). One eye was presented with a neutral grayscale face image, which was generated by averaging different 20 Asian women’s faces, on a gray background, while the other eye was presented with a series of color dynamic Mondrian patterns refurbished at 10 Hz in full screen. The face image subtended 10.2 × 6.3 degrees and was randomly situated on either the left or the right side of the screen. The mean luminances of the face image, the Mondrian patterns, and the background were 17.6, 39.6, and 12.1 cd/m^2^, respectively. The stimuli were presented for 3 s in each trial. Two adult observers have confirmed that the face presented to one eye can be completely suppressed by dynamic Mondrian patterns presented to the other eye during CFS.

### Procedure

The FPL paradigm consisting of 32 trials was used in our experiment. A fixation figure was shown in the center of the monitor accompanied by a short beep sound prior to each trial to attract the infant’s attention. After confirming that the infant was looking at the fixation figure, the experimenter started the trial. In each trial, the stimulus was presented for 3 s. The position of the face image was randomly assigned in each trial. The parents were instructed to close their eyes during the experiment. An observer, who did not know the stimulus identity, judged whether the infant looked at the left half or at the right half of the screen based on an offline video movie. When only “no-looking” was recorded, the trial was excluded. Forty percent of the trials were recorded by a second trained observer. The interrater reliability of the two observers was calculated by intraclass correlation coefficient (ICC) using SPSS statistical package version 23 (ICC = 0.90 with 95% confidence interval = 0.86–0.93).

### Results

**FIGURE 2:**
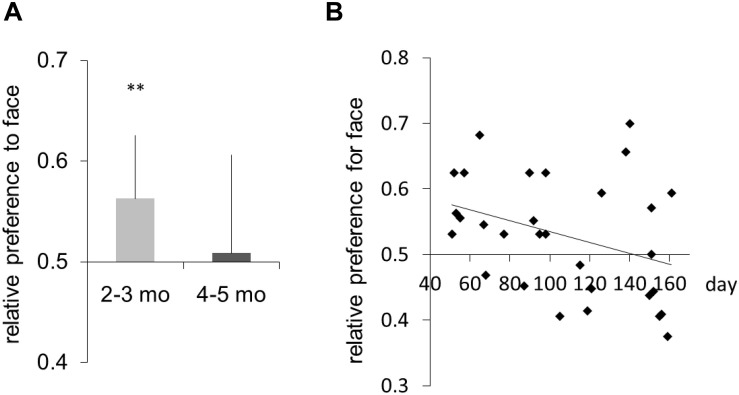
**(A)** Mean relative preference for face image. Error bars are +1 standard error of the mean. **(B)** Individual data showing preference for face image. The horizontal axis represents age in days. The line is the regression line fitted to the individual data. Asterisks indicate the significance level of statistical differences: **p < 0.01.

The mean number of completed trials per participant was 28.58 (SD = 5.58). Preference scores were calculated as the probability of correct judgment for the position of the face image. We regarded these preferences as the detection of the face image. Figure 2A shows the average preference scores for the face image (2- to 3-month-old infants: mean = 0.569, SD = 0.05; 4- to 5-month-old infants: mean = 0.508, SD = 0.09). Two-tailed one-sample *t*-tests against a chance level of 0.5 were conducted for each age group. Significant preference for the face image was observed in the 2- to 3-month-old infants [one-sample *t*-test (vs. chance level, 0.5), *t*(14) = 3.87, *p* < 0.01, *d* = 0.99; a *post hoc* power analysis showed that the study had above 94% power to detect a significant difference at *p* < 0.05] but not 4- to 5-month-old or 5- to 6-month-old infants [*t*(14) = 0.33, n.s.]. An independent *t*-test revealed that the preference scores were different in the two age groups [*t*(28) = 2.11, *p* < 0.05, *d* = 0.77; a *post hoc* power analysis showed that the study had above 80.8% power to detect a significant difference at *p* < 0.05]. Moreover, there was a strong negative correlation between age and preference score (*r* = -0.349, *p* < 0.05; Figure 2B). These results suggest that 2- to 3-month-old infants are sensitive to masked face images that adults cannot perceive. The immature binocular visual system may probably perceive different images from different eyes simultaneously, and the infant may lose this ability after establishing binocular suppression at 4–5 months of age.

It is well known that spatial and temporal contrast sensitivity is significantly lower in infants compared with adults (Teller, 1998). It is possible that the 2- to 3-month-old infants might have been able to detect the face image due to their low sensitivity to the dynamic change in the mask in Experiment 1. Therefore, the dynamic Mondrian patterns in the present study may not have enough intensity to generate interocular suppression. Hence, we tested this possibility in Experiment 2.

## Experiment 2

In Experiment 2, we examined whether the 2- to 3-month-old infants have enough contrast sensitivity to perceive the dynamic Mondrian patterns. A gray background was presented to one eye, while static Mondrian patterns and dynamic Mondrian patterns were presented to the other eye side by side and simultaneously. If the infants had enough contrast sensitivity to perceive the dynamic Mondrian patterns, they would detect the change and show a preference for the side of dynamic Mondrian patterns.

### Participants

Ten infants aged 2–3 months (7 male, 8 female, mean age = 78.9 days, and age range 54–89 days) participated in the study. Although eight other infants were tested in Experiment 2, they were excluded from the analysis because of fussiness (*n* = 6) or side bias of more than 90% (*n* = 2).

### Stimuli

**FIGURE 3:**
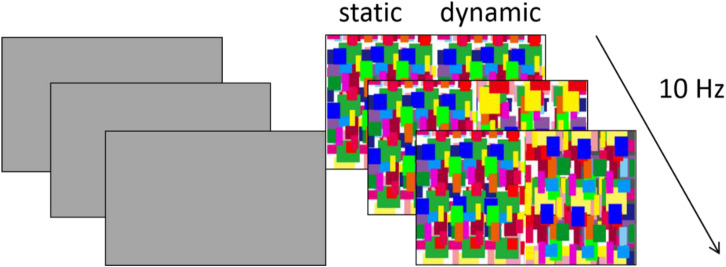
Example of the experiment stimulus in Experiment 2. In each trial, dynamic and static Mondrian masks were presented to one eye, while a gray background was presented to the other eye. The position of the static/dynamic stimulus was randomized.

Two different images were shown dichoptically to both eyes of the infants. One eye was presented with a gray background with a luminance of 17.6 cd/m^2^. The other eye was presented with a half side of dynamic Mondrian patterns, which was identical to that from Experiment 1, and a half side of static Mondrian patterns, which represented one frame of the dynamic Mondrian patterns (Figure 3). The dynamic Mondrian patterns alternated at 10 Hz, while stimuli were presented for 3 s in each trial.

### Apparatus and Procedure

The apparatus and procedure were identical to those used in Experiment 1. Each infant was presented with 32 trials in which the position of the dynamic Mondrian patterns was randomized. Forty percent of the trials were recorded by a second trained observer. The interrater reliability of the two observers was calculated by ICC using SPSS statistical package version 23 (ICC = 0.91 with 95% confidence interval = 0.87–0.95).

## Results

**FIGURE 4:**
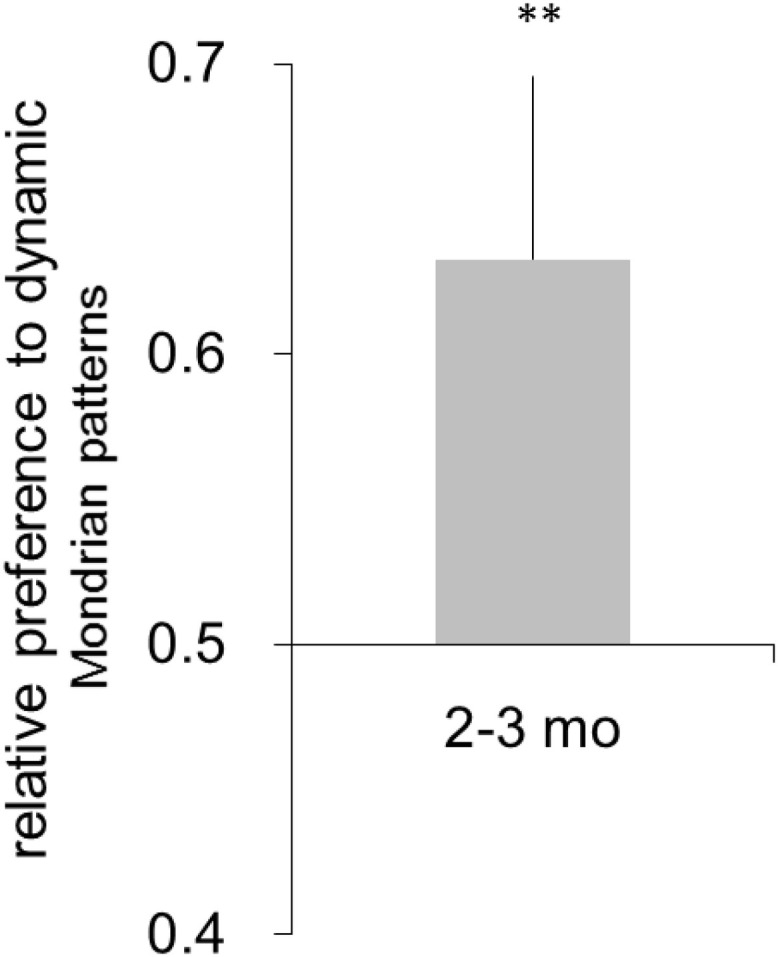
Mean relative preference for the face image. Error bars are +1 standard error of the mean. Asterisks indicate the significance level of statistical differences: **p < 0.01.

The mean number of completed trials per participant was 28.90 (SD = 5.13). Preference for the dynamic Mondrian patterns was observed in the 2- to 3-month-old infants (mean = 0.508, SD = 0.09; Figure 4). A one-sample *t*-test showed that the infants significantly preferred the dynamic Mondrian patterns over chance level [*t*(9) = 4.70, *p* <.01, *d* = 1.57; a *post hoc* power analysis showed that the study had above 99% power to detect a significant difference at *p* < 0.05). These results suggested that 2- to 3-month-old infants could detect the change in the dynamic Mondrian patterns. Therefore, it is unlikely that the detection of the face image by the 2- to 3-month-old infants in Experiment 1 was simply the consequence of their poor spatiotemporal contrast sensitivity.

## General Discussion

The present study investigated the development of binocular suppression among 2- to 5-month-old infants by using the CFS technique. In Experiment 1, we investigated whether infants could perceive a face image masked by dynamic Mondrian patterns. If the function of binocular suppression has not emerged yet, infants should detect the face image and show a significant preference for side where the face image was presented. The results revealed that only the 2- to 3-month-old infants showed a preference for the face side. In Experiment 2, we confirmed that the 2- to 3-month-old infants had enough sensitivity to perceive the change in the dynamic Mondrian patterns. This has confirmed that their ability to detect the masked face image did not stem from weak contrast sensitivity. These results indicated that the immature binocular visual system in 2- to 3-month-old infants may allow them to perceive different images from different eyes simultaneously, while adults’ visual perception would be completely suppressed by the input from the other eye.

The infant may lose the ability to perceive different images from different eyes simultaneously after establishing binocular suppression at 4–5 months of age. Declines in developmental trajectories have been found in many aspects of perception (for a review, see Lewkowicz and Ghazanfar, 2009). For instance, a recent study has reported that 3- to 4-month-old infants react directly to low-level image features that adults might ignore and that this ability would be lost after 5 months of age (Yang et al., 2015). Although some functions show regressive developmental processes, the shifts of the computational scheme reflect the development in the visual system of young infants.

Using CFS, we found different results from those studies (i.e., Brown and Miracle, 2003; Kavšek, 2013b) using preferential looking methods to test the discrimination between fusible and rivalrous patterns. Infants aged 2–3 months old showed a reversal preference for the rivalrous patterns in our study; in contrast, infants aged 2–4 months old preferred the fusible patterns rather than the rivalrous patterns in Brown and Miracle (2003) and Kavšek (2013b). It must be noted that the stimuli were completely different in these studies: both sides of the stimulus were rivalrous patterns in present study, but those in previous studies were fusible patterns vs. rivalrous patterns. Even though we knew that 2- to 3-month-old infants might prefer the fusible pattern from previous studies, it is difficult to predict how infants perceive the rivalrous patterns. It is possible that these infants perceive only one image from one eye at a time like adults. Another possibility is that these infants’ perceived image is an unstable mixture of the two images from two eyes, analogous to the transition state of binocular rivalry in adults, because the immature binocular functioning might not have been sufficient for providing the energy to suppress the inputs from other eye completely. Our results suggest that the latter is more plausible because these infants could detect the face during CFS, indicating that the dynamic Mondrian pattern can only suppress a part of the inputs from the other eye. Therefore, the development of binocular rivalry seems to be a continuous process after birth. The 2- to 3-month-old infants might experience an incomplete form of binocular rivalry, perceiving an unstable mixture from two eyes, and then develop an adult-like binocular rivalry after 3 months of age.

Recently, it has been reported that individuals with autism spectrum disorder (ASD) demonstrated a slower rate of binocular rivalry alternations with longer durations of mixed percepts that matched typically developing infants, which might be caused by the lack of balance between cortical excitation and inhibition (Robertson et al., 2013). The imbalance between cortical excitation and inhibition in young infants may impair interocular suppression, which permits them to perceive the face image under a dynamic Mondrian pattern. Promising future research would be to explore whether newborns who would later be diagnosed with ASD would have a different binocular rivalry or suppression.

In the present study, we found that 2- to 3-month-old infants could perceive the face target during CFS. In addition to the possibility that the immature binocular visual system allows 2- to 3-month-old infants to perceive different images from different eyes simultaneously, subcortical processing could be involved in face detection under binocular suppression in 2- to 3-month-old infants. Previous studies show that newborns can detect faces while the visual cortex is still immature, indicating that subcortical pathways are involved in face detection in infants less than 3 months of age (Johnson et al., 1991; Mondloch et al., 1999; Cassia et al., 2004; Di Giorgio et al., 2012; for a review, see Johnson, 2005). Furthermore, a recent study demonstrated that subcortical face processing affects face detection in 2-month-old infants (Nakano and Nakatani, 2014). On the other hand, functional magnetic resonance imaging in adults revealed that the subcortical region responds to invisible face stimuli under CFS (Jiang and He, 2006; Troiani and Schultz, 2013). Taken together, these pieces of evidence indicate a possibility that the face detection of 2- to 3-month-old infants reflects subcortical processing of the face.

To conclude, the current study provided the first investigation of binocular suppression in infants using CFS. Our findings suggested that infants aged 2–3 months could detect the target under CFS and that by 4 months of age, binocular suppression emerges, resulting consequently in the inability of 4- to 5-month-old infants to perceive the target during CFS.

## Data Availability Statement

The datasets presented in this study are available from the corresponding author, JY, upon reasonable request.

## Ethics Statement

The studies involving human participants were reviewed and approved by Ethical Committee of Chuo University. Written informed consent to participate in this study was provided by the participants’ legal guardian/next of kin.

## Author Contributions

JY developed the study concept. All authors contributed to the study design. JY performed testing and data collection, data analysis and interpretation under the supervision of SK and MY, and drafted the manuscript. SK and MY provided critical revisions. All authors approved the final version of the manuscript for submission.

## Conflict of Interest

The authors declare that the research was conducted in the absence of any commercial or financial relationships that could be construed as a potential conflict of interest.
